# Canine urothelial carcinoma: expression of Periostin in spontaneous canine urothelial carcinoma and its correlation with histological features

**DOI:** 10.3389/fvets.2023.1258247

**Published:** 2023-11-16

**Authors:** Eleonora Brambilla, Rafał Ciaputa, Paola Crepaldi, Stanislaw Dzimira, Marcin Nowak, Piotr Dziegiel, Aleksandra Piotrowska, Veronica Mollica Govoni, Carlos Eduardo Fonseca-Alves, Renée Laufer-Amorim, Damiano Stefanello, Stefano Romussi, Valeria Grieco

**Affiliations:** ^1^Department of Veterinary Medicine and Animal Science, University of Milan, Lodi, Italy; ^2^Division of Pathomorphology and Veterinary Forensics, Department of Pathology, Faculty of Veterinary Medicine, Wroclaw University of Environmental and Life Sciences, Wroclaw, Poland; ^3^Department of Agricultural and Environmental Sciences - Production, Landscape, Agroenergy, University of Milan, Milan, Italy; ^4^Division of Histology and Embryology, Department of Human Morphology and Embryology, Wroclaw Medical University, Wroclaw, Poland; ^5^Department of Veterinary Surgery and Animal Reproduction, Universidade Estadual Paulista (UNESP), Botucatu, Brazil; ^6^Institute of Health Sciences, Universidade Paulista (UNIP), Bauru, Brazil; ^7^Department of Veterinary Clinic, Universidade Estadual Paulista (UNESP), Botucatu, Brazil

**Keywords:** POSTN, dog urothelial carcinoma, urinary bladder, canine urothelial carcinoma, cancer associate fibroblasts (CAFs)

## Abstract

The tumor microenvironment is considered one of the main players in cancer development and progression and may influence the behavior of cancer cells. Periostin (POSTN) is an extracellular matrix protein, and its main functions are induction of fibrillogenesis, fibroblastic cell proliferation and migration, enhancing regeneration in normal tissue, and promoting metastasis in case of neoplasia. POSTN has already been studied in humans in several normal tissues, inflammatory processes, and neoplasms, revealing an important role in tumor progression in various types of cancer, such as colon, lung, head and neck, breast, ovarian, and prostate. In these latter, high levels of POSTN are usually associated with a more aggressive tumor behavior, tumor advanced stages, and poor prognosis, while in human bladder urothelial carcinoma (BUC), unlike in most tumors, POSTN expression seems to be downregulated. The expression of this marker has been poorly investigated in veterinary medicine; thus, this study aimed to immunohistochemically investigate the presence and the intensity of POSTN expression in canine BUCs and to determine a possible relationship between POSTN expression and histopathological features such as mitotic count and muscular and vascular invasions. For the present retrospective study, archived samples from 45 canine BUCs and 6 non-neoplastic canine bladders were considered for histological evaluation and immunohistochemical examination for the expression of POSTN. POSTN expression was semi-quantitatively assessed considering both the percentage of the neoplastic stroma positive for POSTN and the intensity of the immunohistochemical labeling. Histologically, 38 out of 45 tumors were papillary and 7 out of 45 were non-papillary. All tumors were infiltrating, being that 21 were muscle-invasive, and a significant correlation between this feature and vascular invasion emerged (*P* = 0.0001). In normal bladder tissue, as reported in humans, a thick and strongly positive belt of POSTN was visible, and in canine BUCs, stating that the expression is comparable with human benign as well as malignant bladder tissue, a general decrease in POSTN expression was observed except for a strongly labeled ring of POSTN observed around some neoplastic nodules infiltrating the muscle layer. Moreover, POSTN expression and mitotic count were significatively inversely correlated (*P* = 0.0015). The fact that POSTN protein is less expressed in urothelial carcinomas than in the normal bladder supports what was reported in human BUCs and, together with the negative correlation between mitotic count and protein expression that emerged in the present retrospective study, encourages further prospective follow-up studies to verify the possible role of POSTN in canine BUCs as a prognostic marker, and also as a possible target for the development of future anticancer therapies.

## 1 Introduction

The tumor microenvironment can be defined as everything that surrounds tumor cells that are not the tumor cells themselves and is considered nowadays as one of the main players in cancer development and progression (1). The tumor microenvironment is highly complex and consists of non-tumor cells (i.e., cancer-associated fibroblast, endothelial cells, or infiltrating leukocytes) and a large list of proteins and soluble factors such as extracellular matrix (ECM) proteins, and soluble components such as hormones, growth factor, and cytokines (1, 2). The way that microenvironment components interact among them and with the tumor cells is very complex and only partially understood (1, 3).

Tumor cells can alter the microenvironment by changing the properties of the host tissue and vice versa, and the composition of the tumor microenvironment may influence the behavior of cancer cells (2).

Periostin (POSTN) is a non-structural protein of the (ECM), previously named osteoblast-specific factor-2, which was first identified in 1993 as a putative adhesion protein for preosteoblasts in a mouse osteoblastic MC3T3-E1 cell line (4–6). This protein was then named POSTN since it was identified in the periosteum and in periodontal ligament, teeth, and cardiac valves that physiologically undergo mechanical stress during tissue regeneration and development (3). Investigation into heart diseases such as myocardial infarction and cardiac hypertrophy revealed two key POSTN functions. The first one is represented by the induction of fibrillogenesis stimulating type one collagen production and cross-linking and then inducing fibrosis (7). The second main action consists of the activation of cell migration through the integrin binding, which stimulates fibroblastic cell proliferation and migration (8), enhancing regeneration in normal tissue and promoting metastasis in case of neoplasia.

POSTN plays a central role in the homeostasis of normal tissues by regulating cell differentiation and proliferation (1). POSTN is physiologically mainly produced by stromal cells such as myofibroblasts, osteoblasts, and bone marrow-derived mesenchymal stromal cells. POSTN is present in a wide variety of normal adult and fetal tissues, such as embryonic periosteum, periodontal ligament, placenta, cardiac valves, adrenal glands, lung, thyroid, stomach, colon, vagina, ovary, testis, prostate, breast, and urinary bladder (6, 9, 10). In particular, a belt of periostin in the bladder stroma beneath the surface epithelium has been identified (11). POSTN is also produced by cancer-associated fibroblasts (CAFs), and its expression seems to be upregulated in several pathologies, such as inflammation, tissue repair, and malignant transformation (12). Tumor cells, especially cancer stem cells, can also produce POSTN, which has been shown to regulate multiple biological behaviors of tumor cells, including proliferation, survival, invasion, angiogenesis, metastasis, and chemoresistance (13).

In human medicine, studies on several cancer types, such as non-small cell lung cancer (NSCLC), renal cell carcinoma (RCC), malignant pleural mesothelioma (MPM), breast cancers (1, 14, 15), and others, have demonstrated that high POSTN levels are usually associated with a more aggressive tumor behavior, advance stage, and poor prognosis, suggesting that POSTN levels could be a useful prognostic biomarker (1, 9, 16–21).

Unlike in most of these latter tumors, POSTN expression appears to be downregulated in human BUCs compared with normal tissue (11).

The expression of this marker has been poorly investigated in canine-bearing tumors. Only two studies focusing on canine mammary tumors (22, 23), one on squamous cell carcinoma and one on osteosarcoma, are present in the literature (24, 25). One of the two studies regarding canine mammary tumors (22) demonstrated a positive correlation between the expression of POSTN in CAFs in mammary carcinomas, the tumor grade, and the expression of the Ki-67 proliferative antigen, suggesting a role of POSTN in the pathogenesis of canine mammary tumors as in humans. Similarly, in canine squamous cell carcinoma and osteosarcoma, an increased expression of POSTN was detected in the neoplastic stroma.

Bladder cancer comprises 1.5–2% of all naturally occurring cancers in dogs, a rate similar to that reported in humans (26). Dogs with invasive BUCs recently were proposed as a “large animal” model of invasive BUCs in humans because they show similar invasive behavior, morphology, and metastasis location (26).

State of that, this study aimed to immunohistochemically investigate the presence and the intensity of POSTN expression in canine BUCs to determine an eventual relationship between levels of POSTN expression and histopathological features such as tumor type, tumor infiltration, vascular invasion, mitotic count, and tumor grade.

## 2 Materials and methods

### 2.1 Samples and histology

For the present retrospective study, histological slides and related paraffin blocks from 45 cases of canine BUCs and 6 non-neoplastic canine bladders, the latter from necropsied dogs that died from trauma, were retrieved from the archives of the Pathology Unit of the Veterinary Medicine and Animal Sciences Department of the University of Milan, from the archives of the Pathology Department of the Veterinary Faculty of the Wroclaw University of Environmental and Life Sciences, and from the Veterinary Pathology Service of the São Paulo State University in Brazil. BUC samples belonged to 45 dogs. Breed and sex were known in 44 out of 45 cases examined (98%). Breeds were so represented: mixed breed dogs (18) (41%), Yorkshire terriers (3) (7%), poodles (3) (7%), West Highland White terriers (3) (7%), Miniature Schnauzers (2) (5%), Labrador retrievers (2) (5%), Dachshunds (2) (5%), Bernese mountain dogs (2) (5%), Drahthaar (1) (2%), hound (1) (2%), American cocker spaniel (1) (2%), Beagle (1) (2%), Siberian husky (1) (2%), Large Swiss shepherd dog (1) (2%), Bull terrier (1) (2%), and English bulldog (1) (2%). A total of 18 (41%) out of 44 were male dogs (one of which was neutered), while 26 (59%) were female dogs (spayed in 7 cases). The age of the dogs, which was known in 43 out of 45 (96%) cases, ranged from 7 to 17 years with an average of 11 years (1st quartile = 10 years; 3rd quartile = 12 years).

Samples were derived from excisional biopsies (31 cases), cystoscopic biopsies (13 cases), and necropsy (1 case). Biopsies smaller than 5 mm were excluded from the caseload. All samples had been fixed in 10% buffered formalin, and during trimming, a complete longitudinal section was routinely processed for histology. In brief, samples were dehydrated in graded alcohols, clarified in xylene, and embedded in paraffin. From the paraffin block of each sample, serial 5 microns thick sections were obtained. One section was stained with hematoxylin–eosin to be histologically evaluated and classified using the WHO 2004 classification of domestic animal tumors (27). During the histological examination, tumors were graded based on the two more cited grading systems for canine BUCs: Valli et al. (28) and Meuten and Travis (29). Moreover, tumor extension (through the thickness of the bladder), whether it invaded only the chorion or extended to the muscular layer, mitotic count (number of mitoses in 10 high-magnification fields, corresponding to an area of 2.37 mm^2^), and presence of neoplastic cell within vessel lumina (vascular invasion) were recorded.

### 2.2 Immunohistochemistry

Staining was performed on a LEICA BOND-MAX (Leica Biosystems, UK) according to the following protocol. First, tissues were deparaffinized (Bond Dewax Solution, Leica Biosystems, UK) and pre-treated with the Bond Epitope Retrieval Solution 1 (Leica Biosystems, UK) for 20 min. The activity of the endogenous peroxidase was blocked by peroxide block using the BOND Polymer Refine Detection System (Leica Biosystems, UK). A polyclonal antibody directed against human POSTN (NBP1-82472, Novus Biologicals) was used as the primary antibody. The protein BLAST analysis using the human POSTN sequence against the canine database demonstrated 97.76% identity (274 of 377 amino acids), with zero gaps (0%), between the human and canine POSTN sequences, being the genes for *POSTN* (POSTN) conserved in different species including dogs and humans. The anti-POSTN antibody, diluted 1:100 in the Bond Primary antibody Diluent (Leica Biosystems, UK), was applied for 15 min at room temperature. Next, the samples were incubated with Post Primary and Polymer using the BOND Polymer Refine Detection System (Leica Biosystems, UK). The 3,3-diaminobenzidine (DAB Chromogen) was applied as the substrate for the reaction, and then all the sections were counterstained with Hematoxylin (BOND Polymer Refine Detection System, Leica Biosystems, UK). A section of a canine mammary tumor was used as a positive control, while negative controls were obtained by substituting the primary antibody with rabbit normal serum at the same protein concentration as the primary antibody.

Immunohistochemical results were assessed within each complete longitudinal tumor section using a semiquantitative score (Table 1), considering the percentage of tumor stroma positive for POSTN in relation to the total tumor stroma included in the complete longitudinal tumor section. Furthermore, the intensity of the immunohistochemical labeling was defined as absent (0 points), weak (1 point), moderate (2 points), and intense (3 points). Where the signal intensity was not homogeneous, the percentage of tumor stroma expressing POSTN with the highest intensity was considered.

**Table 1:** The semiquantitative score applied for the assessment of the immunohistochemical extension and intensity of the expression of POSTN in the tumor stroma.

**Percentage of neoplastic stroma positive for POSTN**	**Points**
0	0
1–5%	1
6–20%	2
21–50%	3
51–60%	4
>60%	5

### 2.3 Statistical analysis

Statistical analysis was performed by JMP. We calculated the descriptive statistics (absolute and relative frequencies) of the categorical variables collected as breed, sex, tumor type, muscular infiltration, vascular invasion, tumor grading, and POSTN expression score. We also described the distribution of continuous variables such as age and mitotic count with the average + SD and/or first and third quartile for age and mitotic count. The goodness of fit to a normal distribution was assessed for the mitotic count with the Shapiro–Wilk W-test. The presence of a significant relationship between categorical variables (i.e., type of tumor—infiltrating or not infiltrating lamina propria, muscular invasion—present or absent, and vascular invasion—present or absent) was then analyzed with the Pearson and exact chi-square tests to evaluate the presence of a significant relationship. Similarly, the presence of any statistical relationship between the extent of the immunohistochemical signal with the intensity in terms of score and any correlation of the two parameters with the categorical variables above was assessed.

To evaluate the effect of the immunohistochemical POSTN expression score, as a categorical variable with four levels, on the continuous variable mitotic count, a one-way ANOVA model was used after checking the assumption of normality for the mitotic count variable using the Shapiro–Wilk test, and the assumption of equal variances using the Levene test.

## 3 Results

### 3.1 Histology

According to the last WHO (2004) classification of domestic animal tumors in use, 38 tumors were diagnosed as papillary (84%) and 7 (16%) cases as non-papillary (30).

All tumors had an infiltrative growth pattern, but the infiltration of the surrounding tissue occurred at different levels: in 12 out of 45 (27%), it was limited to the bladder lamina propria, while in 21 out of 45 (47%), the tumor extended to the muscle layer. However, in other 12 out of 45 (27%) cases, the depth of the infiltration was not completely evaluated since samples (being collected as cystoscopic biopsies) lacked the muscle layer.

Aggregates of neoplastic cells in the vessel lumina (vascular invasion) were detectable in 26 out of 45 (58%) neoplastic cases. In 7 of these 26 cases, the tumor infiltrated only the lamina propria; in 18 cases, the neoplasm also involved the muscle layer; while in one case, the depth of tumor extension was not evaluable.

In the 38 papillary BUCs, papillary or cauliflower-like structures projecting into the bladder lumen were visible. These projections showed a central fibrous stalk, varying in thickness, covered by multiple layers of neoplastic urothelium characterized by mild-to-severe cellular atypia. Secondary or branching villous projections from the main tumor emerged in advanced tumors.

Non-papillary and infiltrating BUCs appeared as plaques, raised masses, or flat nodules. These tumors were often variably ulcerated and, in all seven cases, infiltrated into the deeper muscle layers.

**Figure 1:**
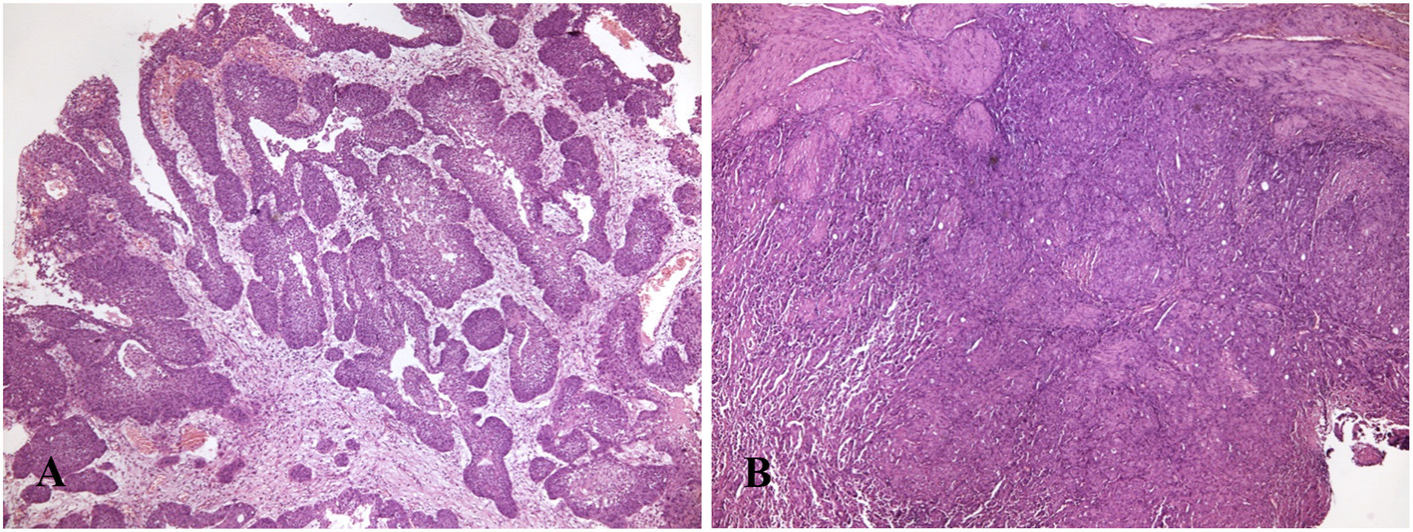
**(A)** Dog with papillary and infiltrating urothelial carcinoma. Papillary projections supported by a central fibrous stalk, varying in thickness, are covered by multiple layers of neoplastic urothelium (H&E, 40X). **(B)** Dog, urinary bladder with non-papillary and infiltrating carcinoma appeared as a thick plaque (H&E, 40X).

In both papillary and non-papillary tumors (Figure 1), neoplastic cells were polygonal, with sharp cell borders, a variable amount of eosinophilic cytoplasm, characterized by the presence of colorless to eosinophilic cytoplasmic inclusions called Melamed–Wolinska bodies (27, 29).

The round to oval nuclei were generally large and vesicular, and nucleoli were often prominent. Varying degrees of differentiation and anaplasia were present, and atypical nuclei were frequently observed. Mitoses ranged from 4 to 73 in 2.37 mm^2^, averaging 22.5. Bizarre mitotic figures were variably seen in tumors with a high mitotic count. Within the tumor, limited areas of squamous and/or glandular metaplasia and desmoplastic reaction were variably observed (27, 29).

Tumors were often characterized by superficial ulceration and areas of coagulative necrosis and multifocal hemorrhages variably in width; moreover, BUC‘s lamina propria and submucosa were frequently infiltrated by numerous small mature lymphocytes and plasma cells scattered or variably arranged in aggregates or follicular structures.

Based on the Valli et al. (28) grading system, which considers the nuclear shape and position, the appearance of the chromatin, and the presence of prominent nucleoli, 11 out of 45 (24%) tumors were graded as grade 1, 26 out of 45 (58%) as grade 2, and 8 out of 45 (18%) tumors as grade 3.

Based on the Meuten and Travis (29) grading system, which also focuses on the invasiveness of the tumor, considering invasive tumors as high grade and non-invasive tumors as low grade, all the tumors (100%) were graded as high grade.

### 3.2 Immunohistochemistry

**Figure 2:**
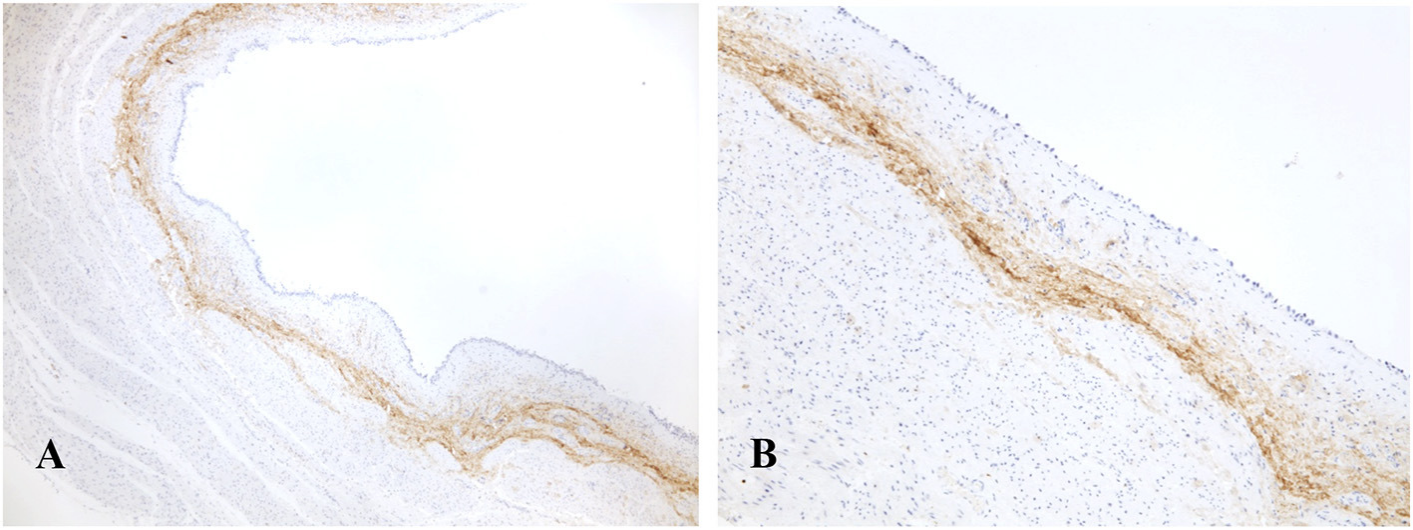
Normal urinary bladder of a dog. The immunohistochemical labeling for POSTN is constantly and extensively visible as a compact intensely positive belt located in the supporting connective tissue beneath the urothelium [Immunohistochemistry: **(A)** 40X, **(B)** 100X].

In the three non-neoplastic bladders examined, POSTN was intensely and diffusely (>60%) expressed (Table 1) within the connective tissue of the lamina propria and submucosa just beneath the bladder epithelium (Figure 2).

**Figure 3:**
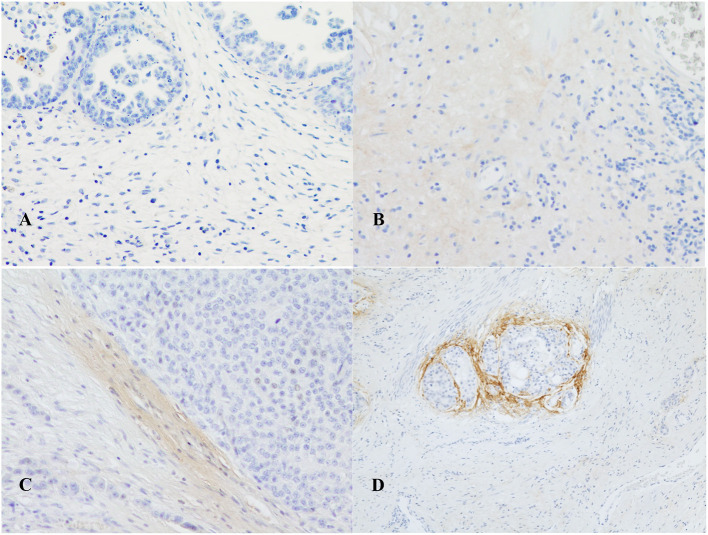
Immunohistochemical expression of periostin in canine urothelial carcinomas: **(A)** absence of expression; **(B)** weak; **(C)** moderate (40X); and **(D)** intense (20X). In **(D)**, an intense ring of POSTN was visible around some groups of neoplastic cells infiltrating the urinary bladder muscle layer.

Conversely, the extension of the POSTN expression in BUCs generally ranged from 0% to 60% (0 to 4 points, Table 2) and, when present, it was haphazardly localized in the neoplastic stroma and varied in intensity from weak to intense (0 to 3 points, Table 3). In 5 out of 45 (11%) tumors, POSTN was absent (0 points). In 8 out of 45 (18%) cases, the extension of the immunohistochemical label was from 0 to 5% (1 point) and, among these latter, in 6 cases, the intensity was weak (1 point), and in two was moderate (2 points). Extension of the POSTN expression ranged from 6 to 20% in 16 (36%) cases (2 points), 9 with weak intensity (1 point) and 7 with moderate intensity (2 points). Moreover, an intense ring of POSTN was visible around some groups of neoplastic cells infiltrating the urinary bladder muscle layer (Figure 3).

**Table 2:** POSTN expression extension—immunohistochemical results.

**Number of cases**	**Percentage of neoplastic stroma positive for POSTN**	**Points**
5	0	0
8	1–5%	1
16	6–20%	2
13	21–50%	3
3	51–60%	4
0	>60%	5

**Table 3:** POSTN expression intensity—immunohistochemical results.

**Points**	**Intensity**	**Number of cases**
0	Absent	5
1	Weak	22
2	Moderate	17
3	Intense	1

### 3.3 Statistical relationships between histological variables and POSTN expression

**Figure 4:**
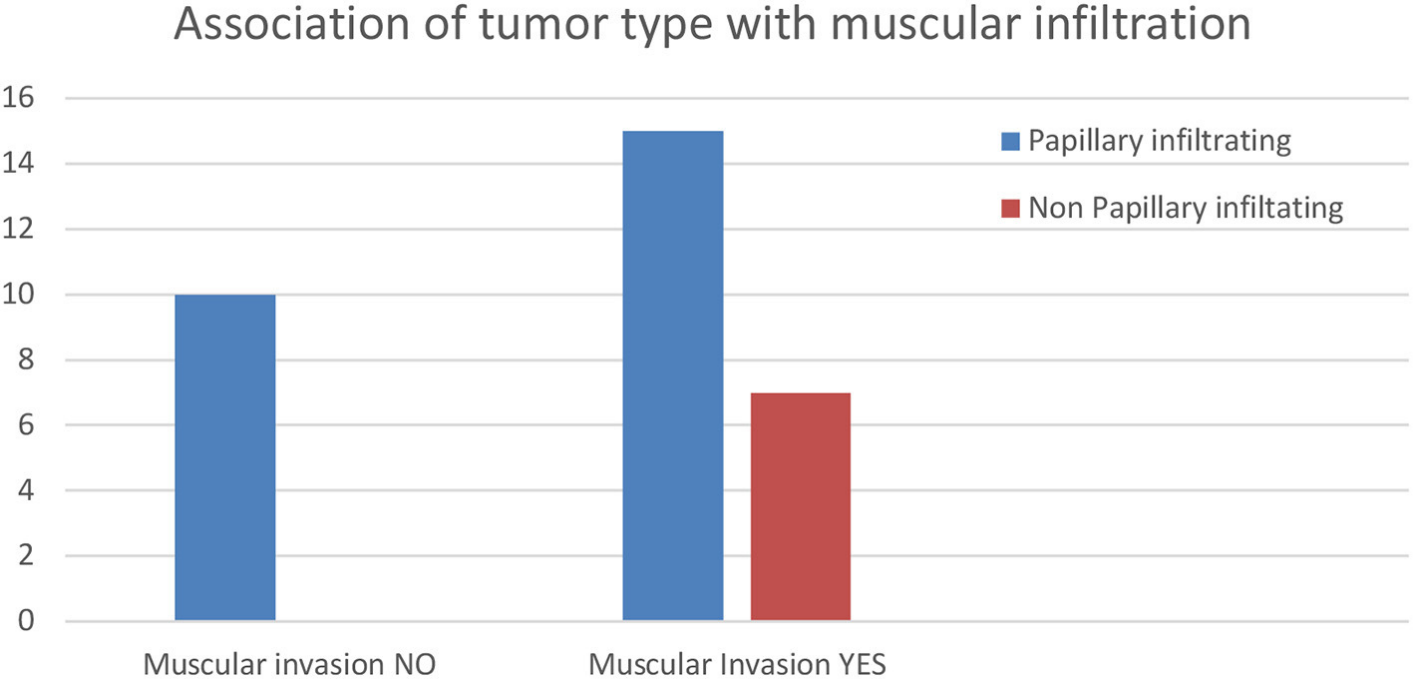
Histogram of the results of the association of tumor type with muscular infiltration.

For the 33 BUCs, including muscle in the histological samples, a significant (*P* = 0.04) relationship between tumor histological type and muscular invasion was observed. Non-papillary infiltrating tumors were all muscle-invasive, while in the case of papillary infiltrating BUCs, 60% of tumors invaded muscle and 40% did not (Figure 4).

**Figure 5:**
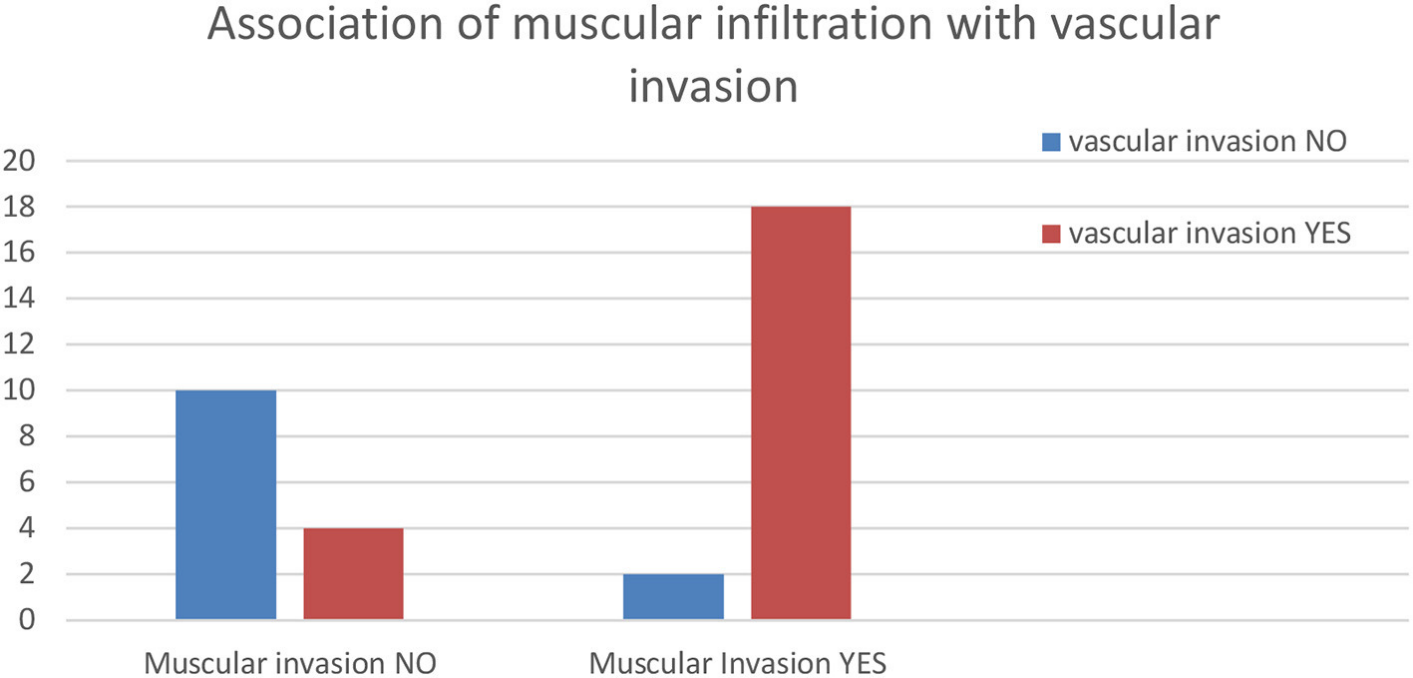
A histogram of the results of the association of muscular infiltration with vascular invasion.

The Chi-squared test showed a significant relationship (*P* = 0.0001) between muscular infiltration and the presence of neoplastic aggregates in the vessel lumina (interpreted as vascular invasion). BUCs with muscular infiltration and vascular invasion (in blue in the right column of the mosaic plot reported above—Figure 5) were significantly more than expected. On the contrary, tumors with no muscular infiltration and no vascular invasion were less than expected (Figure 5).

**Figure 6:**
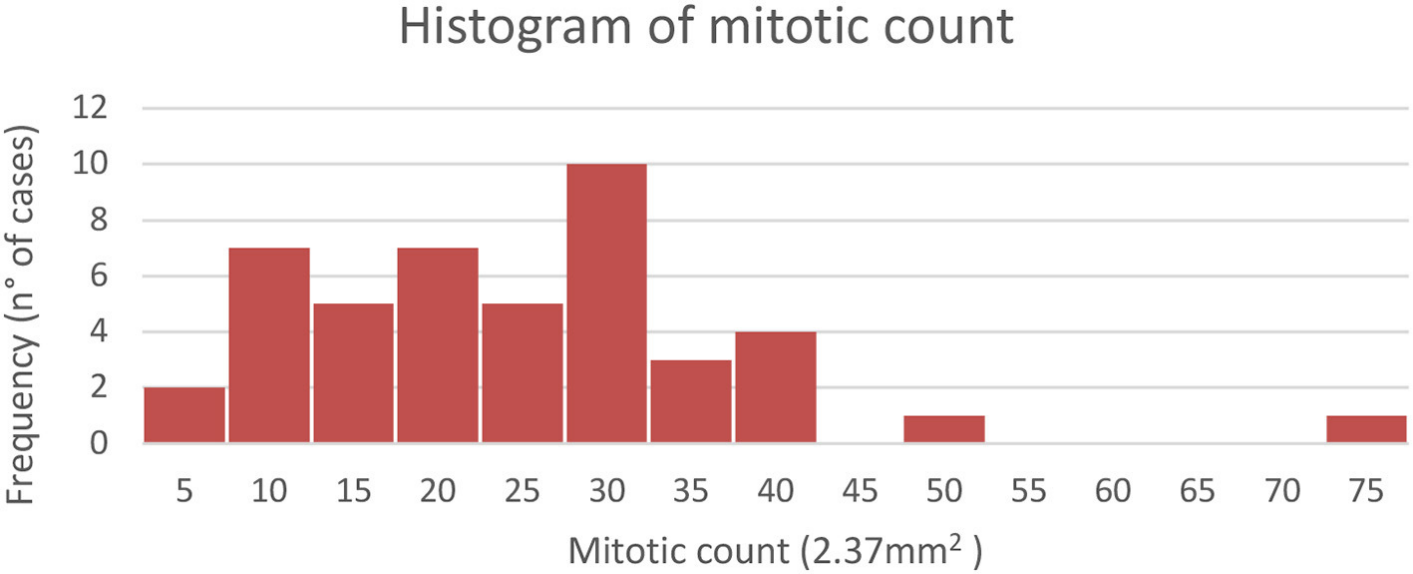
Distribution of mitotic count in the analyzed canine urothelial carcinomas *n* = 45.

The histogram of mitotic counts is reported in Figure 6. Mitoses ranged from 4 to 73 in 2.37 mm^2^, with an average of 22.5. The histogram shows, among the highest frequencies, that the number of mitoses was 10 or 20 in 2.37 mm^2^ in seven cases, while in 10 cases, 30 mitoses were counted. The mitotic count was normally distributed with an average ± SD of 21.4 ± 10.7 mitotic count in the Shapiro–Wilk test, excluding an outlier with 73 mitoses in 2.37 mm^2^ in one dog with a non-infiltrating tumor.

The ANOVA model (*n* = 45, r^2^ = 0.35) of mitotic count with the extension of the immunohistochemical expression of POSTN in BUC stroma (score—Table 1) shows a statistically significant (*P* = 0.0015) mitotic count score, so mitotic count is negatively correlated with increased extension of POSTN expression. Tukey-Kramer's HSD *post-hoc* test highlighted that score 3 had a significantly lower average of mitotic count equal to 12.3 with respect to the average of the scores 0, 1, and 2 that showed an average, respectively, equal to 37, 26, and 25 mitoses. The score 4 showed an average of 19.3, not statistically different from the other three score levels.

Conversely, the Chi-square analysis to verify a possible association between the results obtained with the extension of the immunohistochemical expression of POSTN in BUC stroma and its intensity or with other categorical variables, such as the presence of vascular invasion or muscle infiltration, did not show any significant results.

Moreover, no relationship was observed between the intensity and extension of the POSTN immunohistochemical labeling.

## 4 Discussion

POSTN regulates multiple biological behaviors of tumor cells, including proliferation, survival, invasion, angiogenesis, metastasis, and chemoresistance. Moreover, its expression seems associated with aggressive tumor behavior, advanced stage, and poor prognosis in most human tumors (1, 9, 15–20), except for BUCs in which POSTN seems to be downregulated (21). Since the growing importance of canine BUC as a model for human disease, and since POSTN has been poorly investigated in veterinary medicine, in the present study, POSTN immunohistochemical expression was evaluated in 45 spontaneous canine BUCs.

Considering patients, four of them were from predisposed breeds, such as West Highland White terriers (3 cases) and Beagle (1 case) (31, 32), while the remaining patients belonged to other breeds or, in 18 cases, were mixed-breed dogs. These findings are in line with recent research (13), which suggested that, independently from the predisposed breed, other factors, such as the possible mutation of the *BRAF* gene and epigenetic factors, can be involved in urinary bladder carcinogenesis.

On the other hand, the 2-fold higher BUC risk in spayed/neutered dogs, if compared with intact dogs (33–35), was not confirmed in the present study. These findings deserve to be further investigated on a larger number of samples, especially spayed dogs.

Regarding cell morphology, varying degrees of differentiation and anaplasia were present, and atypical nuclei were frequently observed reflecting what is reported in the literature (29). Mitoses ranged from 4 to 15 in 14 out of 45 (31%) cases, from 16 to 30 in 22 out of 45 (49%) cases, and were more than 30 in 9 out of 45 (20%) cases, reaching 73 mitoses in one non-infiltrating tumor. The mitotic count in 31 (69%) cases was >15 mitoses, which demonstrates that BUCs have a particularly high replicative potential which could indicate a consequent high malignancy of these tumors.

Moreover, all tumors were infiltrating, and their growth pattern was papillary or not papillary in 38 (84%) and seven cases, respectively (16%). These findings are in line with the literature stating that the most common BUC variant in canine species is the papillary and infiltrating one (27, 31). Regarding tumor histological classification and extension, all the non-papillary tumors were muscular invasive, revealing that non-papillary tumors are more prone to invade the muscle layer. Moreover, the Chi-square analysis revealed a significant correlation between muscular invasion and the presence of neoplastic vascular emboli. A recent meta-analysis reviewing several studies on both human and canine BUCs revealed that in most studies on canine BUC, histologically important features such as muscular or vascular invasion were not assessed. In addition, the assessment of tumor local invasion in dogs with BUC has not already been standardized (35). In humans, the muscle-invasive BUC represents only 30% of the cases (36), while in canine patients, most BUCs are invasive, reaching the muscle layer and often invading the lymphatic vessels or even affecting adjacent organs such as the urethra and prostate (29). This is probably due to the advanced stage of the disease at the time of diagnosis (28, 31, 37, 38). Even if, in the present study, all BUCs examined had an infiltrative growth pattern, in the 33 samples in which the muscle layer was also evaluable, the level of the infiltration was limited to the bladder lamina propria in 12 out of 45 (27%) while the tumor extended to the muscle layer of the urinary bladder wall in 21 out of 45 (47%) cases. This is an important finding to be considered in future prospective studies because non-invasive diagnostic tools, nowadays used also in veterinary, such as ultrasound, could contribute to discovering tumors before a deep invasion, and prognosis could be different depending on tumor local extension (chorion or muscle).

However, in other 12 out of 45 (27%) cases, the depth of the infiltration was not completely evaluable, reflecting the fact that the biopsies collected by cystoscopy frequently do not include all muscle layers, making impossible, in most cases, the assessment of local tumor invasion (35, 39).

Another interesting histological feature was the presence of aggregates of neoplastic cells in the vessel lumina (vascular invasion) that was statistically associated with the extension of the tumor in the bladder muscle layer and observed in 58% of the tumors examined. The distinction between pseudo-vascular invasion and true vascular invasion has been assessed based on the identification of criteria proposed by Meuten et al. (40). Namely, at least one of the following criteria is required: the most important criteria used to define vascular invasion in human tumors are thrombus adherent to the intravascular tumor, tumor cells invading through the vessel wall and endothelium, and neoplastic cells within a space lined by lymphatic or blood vascular endothelium. Our results confirm that vascular invasion is a frequent finding in canine BUCs, which are frequently diagnosed at a late stage of development. Noteworthy, a recent study highlighted vascular invasion as a significant indicator of poor patient outcomes in canine BUCs (35); thus, this important finding deserves to be included in the pathology diagnostic report, which could also include the tumor grade.

However, although various grading systems have been formulated for canine BUCs over the decades, none of them is routinely used in veterinary pathology, and the two main grading systems, proposed by Valli et al. (28) and by Meuten and Travis (29), are still not validated with prognostic studies in veterinary medicine (30). However, both these latter grading systems were applied to the tumor included in the present study, and, based on Valli's grading system, 11 out of 45 (24%) tumors were graded as grade 1, 26 out of 45 (58%) as grade 2, and 8 out of 45 (18%) tumors as grade 3. These findings are in line with what has already been reported in the literature (30), indicating that the use of three-tier grading systems, such as that proposed by Valli et al. (28), causes the inclusion of most tumors in grade 2, the intermediate grade.

On the other hand, Meuten's two-tier system application showed that all tumors were high-grade, as they were all infiltrating. Nevertheless, this latter grading system does not consider the level of the tumor extension, and infiltrating neoplasm are included in high grade independently from their extension level, which can be limited to the bladder chorion or involve the deep muscle. In any case, the inclusion of all examined tumors in high grade following Meuten's grading system and the inclusion in grade 2 of most of them when Valli's grading system was applied demonstrates that further studies, considering a larger number of patients and related follow-up, are required to individuate the best grading system for canine BUCs.

However, in human medicine, tumor histological appearance and extension are not the only features considered, and, frequently, immunohistochemical markers are also employed to better characterize the neoplasm behavior and aggressiveness. The present study is the first to evaluate the expression of POTN in the normal canine urinary bladder and BUC, which represent an important tumor in this species and has been suggested as a possible animal model for studying the human counterpart. Regarding the expression of POSTN in the normal canine bladder, in our study, it was constantly and extensively present and was visible as a compact intensely positive belt located in the supporting connective tissue beneath the luminal urothelium. These findings consistently reflect what has been observed in humans and represent an interesting comparative feature demonstrating structural similarities between the canine and human urinary bladder (21). In all neoplastic specimens, the normal POSTN front was interrupted by the tumor growth, but it was still visible in the adjacent non-neoplastic mucosal portions of the bladder.

As far as tumors are concerned, in the neoplastic stroma, POSTN was always less extensively and less intensely expressed than in the normal bladder, and it was absent in 6 cases. Also, these findings align with what Kim et al. (41) reported for human urothelial carcinoma cell lines in which a general decrease in POSTN expression was reported compared to normal bladder tissue. Kim et al. (41) also demonstrated that inhibition of AKT (protein kinase B) by POSTN induced the upregulation of E-cadherin and suppressed the invasiveness of bladder cancer cells (21, 42), hypothesizing that, conversely, the POSTN downregulation could be involved in cancer progression. On the other hand, Silvers et al. (43), performing quantitative real-time PCR in 10 human bladder cell lines, observed high POSTN expression in muscle-invasive bladder cancer cell lines J82, TCC-SUP, and UMUC3.

This is an interesting finding and could reflect what was noted in our samples that had a generally weak or absent expression of POSTN but in which an intense ring of POSTN was visible around some group of neoplastic cells infiltrating the urinary bladder muscle layer. This feature, suggesting a possible protective role of POSTN secreted by CAFs with the aim of containing the neoplastic infiltration, was also noted in a recent study on human urothelial carcinomas, even if the research was focused on the tumor affecting the upper urinary tract (42).

The statistical analysis did not reveal any correlation between the extent and intensity of stromal positivity for POSTN and between these two variables and categorical variables such as type of tumor, tumor infiltration, and muscular and vascular invasion.

However, the statistical analysis demonstrates that POSTN expression and mitotic count were significatively inversely correlated. Although score 3 was defined by the highest range of stromal POSTN expression (21–50%), an influence of this range on the mitotic analysis did not emerge. The interesting relation between POSTN expression and mitotic count, emerging for the first time, was not checked in human BUCs and deserves to be investigated in further studies since it seems to suggest a relationship between POSTN expression and a greater rate of neoplastic cellular proliferation (negative prognostic factor). Mitotic count, although a negative prognostic factor, is, however, not considered a distinguishing criterion for histological grading, so there are no cutoffs for mitotic count in veterinary medicine any more than there are for human medicine (30). In a recent study on human urothelial carcinoma of the urinary tract, the statistical relationships with periostin expression and epidemiological and histological features have been investigated, revealing that there was no significant difference in the mean age, sex, tumor laterality, or histological grade between patients with low and high stromal periostin expression, while the frequencies of tumors with non-papillary gross findings were significantly higher in patients with high stromal periostin expression than in those with low expression (42). The latter correlation between increased periostin expression and papillary tumor morphology is not confirmed by the present study.

On the other hand, the lack of statistical correlation between POSTN expression and tumor grading could instead be due to an inadequacy of the BUC canine grading systems, as already highlighted (31).

From statistical evaluations, the presence of neoplastic emboli does not seem influenced by the expression of POSTN. This finding is not surprising because, as described in the literature, vascular invasion seems to be correlated to other factors, such as complex interactions among tumor cells, stromal cells, and endothelial cells. Many molecules are involved in this cellular crosstalk, including growth factors, cell surface receptors, and cytokines, as described particularly in human breast cancer (43–46). Another aspect observed histologically was the inflammatory infiltrates, especially lymphoplasmacytic, often present within the stroma of the tumors included in the study as described in the literature (28, 29). However, these inflammatory infiltrates were never associated with a variation in POSTN expression compared to the remaining tumor stroma. Moreover, POSTN was arranged, in some cases, to surround muscular infiltrates. These features suggest that POSTN, involved in the biochemical mechanisms of collagen synthesis and fibrillogenesis, could be produced by cancer-associated fibroblasts (CAFs) with a protective purpose against tumor infiltration in deeper layers and vascular invasion.

## 5 Conclusion

In conclusion, in canine BUCs, a general decrease in stromal POSTN expression was observed compared to the normal bladder, and POSTN levels were significantly inversely proportional to mitotic count, suggesting a relationship with a greater rate of neoplastic cellular proliferation (negative prognostic factor). Moreover, as for human BUCs, in some cases examined, a ring of POSTN was observed surrounding the muscle-invasive neoplastic infiltrates. As the extent of POSTN expression inversely correlates with mitotic count, this protein could have a role as a prognostic marker. However, this should be verified in future follow-up studies.

## Data availability statement

The original contributions presented in the study are included in the article/Supplementary material, further inquiries can be directed to the corresponding author.

## Ethics statement

The animal studies were approved by the Comitato Etico Tutela Degli Animali Dell'universita' Degli Studi di Milano (Dipartimento di Medicina Veterinaria e Scienze Animali). The studies were conducted in accordance with the local legislation and institutional requirements. Written informed consent was obtained from the owners for the participation of their animals in this study.

## Author contributions

EB: Conceptualization, Data curation, Methodology, Writing—original draft. RC: Conceptualization, Data curation, Methodology, Writing—review & editing. PC: Formal analysis, Writing—original draft. SD: Conceptualization, Writing—review & editing. MN: Conceptualization, Writing—review & editing. PD: Conceptualization, Writing—review & editing. AP: Methodology, Writing—review & editing. VM: Writing—review & editing, Conceptualization. CF-A: Conceptualization, Writing—review & editing. RL-A: Conceptualization, Writing—review & editing. DS: Data curation, Writing—review & editing. SR: Data curation, Writing—review & editing. VG: Conceptualization, Methodology, Supervision, Writing—review & editing.
